# Tartaric Acid Used for Aiding the Solution of Di-Sulphate of Quinia

**Published:** 1852-06

**Authors:** 


*Tartaric Acid used for aiding the solution of Disulphate of Quinine.*

PATIENTS often object to taking quinine on account of its disagreeable, bitter taste. The following, from the *London Lancet*, may enable physicians to obviate this objection:

A few drops of dilute sulphuric acid will, as is well known, aid to dissolve disulphate of quinine, but the taste of the mixture is then very bitter and disagreeable. Messrs. Bouchardat, Righini, and Ruspini advise tartaric acid to be used for the purpose of hastening the solution of the quinine instead of sulphuric acid; and M. Casorati has found that one grain of the tartaric acid is sufficient to saturate three of disulphate of quinine, the compound being then a sulpho-tartrate of quinine, and not at all unpleasant to the taste.

